# Development of Second Generation Activity-Based Chemical Probes for Sirtuins

**DOI:** 10.3390/molecules26010011

**Published:** 2020-12-22

**Authors:** Alyson M. Curry, Elizabeth Barton, Wenjia Kang, Daniel V. Mongeluzi, Yana Cen

**Affiliations:** 1 Department of Medicinal Chemistry, Virginia Commonwealth University, Richmond, VA 23219, USA; curryam@vcu.edu (A.M.C.); bartonej2@mymail.vcu.edu (E.B.); kangw2@vcu.edu (W.K.); mongeluzid@mymail.vcu.edu (D.V.M.); 2 Institute for Structural Biology, Drug Discovery and Development, Virginia Commonwealth University, Richmond, VA 23219, USA

**Keywords:** sirtuin, activity-based, chemical probes

## Abstract

NAD^+^ (nicotinamide adenine dinucleotide)-dependent protein deacylases, namely, the sirtuins, are important cell adaptor proteins that alter cell physiology in response to low calorie conditions. They are thought to mediate the beneficial effects of calorie restriction to extend longevity and improve health profiles. Novel chemical probes are highly desired for a better understanding of sirtuin’s roles in various biological processes. We developed a group of remarkably simple activity-based chemical probes for the investigation of active sirtuin content in complex native proteomes. These probes harbor a thioacyllysine warhead, a diazirine photoaffinity tag, as well as a terminal alkyne bioorthogonal functional group. Compared to their benzophenone-containing counterparts, these new probes demonstrated improved labeling efficiency and sensitivity, shortened irradiation time, and reduced background signal. They were applied to the labeling of individual recombinant proteins, protein mixtures, and whole cell lysate. These cell permeable small molecule probes also enabled the cellular imaging of sirtuin activity change. Taken together, our study provides new chemical biology tools and future drug discovery strategies for perturbing the activity of different sirtuin isoforms.

## 1. Introduction

Sirtuins are a family of deacylases that utilize NAD^+^ as the co-substrate to remove acyl groups from lysine residues of histones or other cellular protein targets [1,2,3]. Seven mammalian sirtuins have been identified (SIRT1–7) with distinct subcellular localizations and biological functions [4,5,6]. They have been implicated in the regulation of a myriad of cellular events including DNA repair [7], gene silencing [8], mitochondrial biogenesis [9], apoptosis [10], and cell cycle control [11]. Accumulating data suggest that the endogenous sirtuin activities are tightly regulated through compartmentalization [6], post-translational modifications (PTMs) [12], protein–protein interactions [13], as well as NAD^+^ metabolism [14]. In spite of the intense interest in pursuing sirtuins as therapeutic targets [1,15], the connections between sirtuin activity and disease pathogenesis remain elusive. Contemporary molecular biology and proteomic techniques measure protein expression level or abundance, which are indirect estimates of enzymatic activity. The need for innovative chemical probes to evaluate sirtuin activity in native biological matrix becomes apparent.

Functional interrogation of enzymes in complex native proteomes can be accomplished with activity-based chemical probes (ABPs). ABPs are active site-directed small molecules that can directly confer the functional state of a specific enzyme in a complex biological sample [16,17]. Most ABPs comprise three major components: a reactive group (or “warhead”) that targets the active site of an enzyme and forms a covalent linkage with the amino acid residue in the active site; a tag that allows the visualization (fluorophore) or affinity enrichment (biotin) of the active enzyme; and a linker that tethers the warhead with the tag. The past two decades have witnessed an ever-growing interest in developing ABPs for functional proteomics analysis [16,17,18,19]. These ABPs greatly expanded our scope of the “druggable” proteomes and led to the discovery of some potent and selective enzyme inhibitors [17,20].

**Figure 1:**
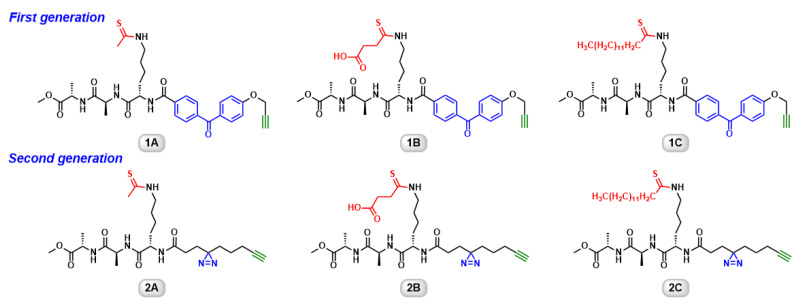
Chemical structures of activity-based chemical probes.

Recently, we developed a group of ABPs for human sirtuins (Figure 1, probes 1A–1C) [21,22]. They feature a thioacyllysine “warhead”, a benzophenone photoaffinity group as well as a terminal alkyne bioorthogonal tag. Thioacyllysine peptides are classified as “suicide substrates” [21,23,24,25,26,27,28,29]. They react with NAD^+^ in the sirtuin active site to form a stalled thioimidate intermediate, leading to mechanism-based sirtuin inhibition [26,30,31]. The introduction of photoactivatable benzophenone converts the non-covalent protein–small molecule interaction into irreversible covalent linkage. The terminal alkyne enables “click” conjugation of fluorophore for the detection or biotin for affinity capture. The first generation of ABPs demonstrate the isoform-selective labeling of recombinant proteins, protein mixtures, and cell lysates [21]. Despite the initial success, there is still room for improvement. For example, the bulky benzophenone may hinder the interactions between the ABPs and target enzymes, causing reduced or loss of labeling. On a related note, the long irradiation time used for benzophenone-containing probes prevents them from being applied to cellular imaging because exposure to intense UV irradiation may result in DNA damage, which could trigger protein mis-translocation or even lethality in cells [32,33].

Herein, we report the synthesis, characterization, and applications of the diazirine-based ABPs for human sirtuins, taking advantage of its small size, chemical stability, under physiological conditions, and high reactivity of the carbene species generated upon photo irradiation [34]. These new ABPs showed significantly improved labeling efficiency, shortened irradiation time, and reduced non-specific labeling compared with the first generation benzophenone-based ABPs. Furthermore, these cell permeable small molecule probes also allowed an evaluation of sirtuin activity change in the cellular setting. Our study provides new chemical biology tools and future drug discovery strategies for perturbing the activity of all sirtuin isoforms.

## 2. Results and Discussion

### 2.1. Design, Synthesis, and Characterization of Diazirine-Containing ABPs

**Figure 2:**
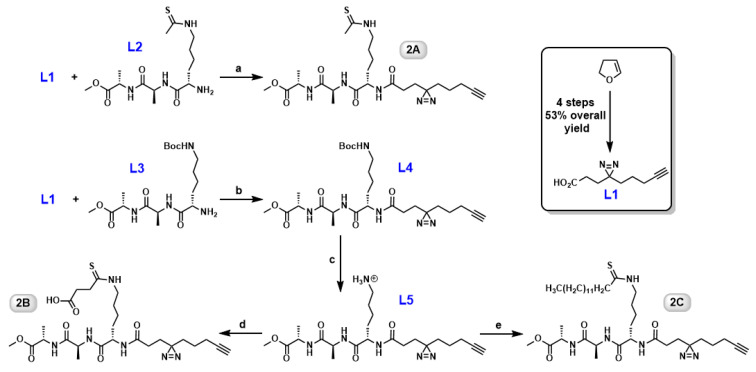
Synthesis of diazirine-based ABPs. **a**. PyBOP, DIPEA, DMF (85%); **b**. PyBOP, DIPEA, DMF (81%); **c**. TFA, triethylsilane, CH_2_Cl_2_ (quantitatively); **d**. tert-butyl thiophenyl mono-thiosuccinate, 5% Na_2_CO_3_, MeOH, 0 °C to r.t. (83%); **e**. thiophenyl thiomyristate, 5% Na_2_CO_3_, MeOH, 0 °C to r.t. (91%).

In the previous study [21], benzophenone was chosen as the photoaffinity group because of its synthetic easiness. However, this bulky substituent is likely to cause steric interference between the probe and the target protein if placed incorrectly [35]. By reducing the size of the photoaffinity group, we may uncover probes with preference for different sirtuin isoforms and improve labeling efficiency as well. The second generation ABPs inherited the lysine–alanine–alanine backbone, but included a diazirine tag instead. Our effort focused on incorporating the diazirine in the later stage of the synthesis (Figure 2). Intermediate L1 was obtained from commercially available 2,3-dihydrofuran in 4 steps with 53% overall yield [36,37]. Tripeptide L2 is prepared routinely in our lab on a gram scale [21]. Then, the carboxylic acid group in L1 and free amino group in L2 were conjugated together through PyBOP-mediated amide bond formation to afford probe 2A (Figure 2). *N*(ε)-Boc-protected tripeptide L3 [21] was used as the common precursor for both probes 2B and 2C. The amide coupling between L1 and L3 resulted in the formation of a more advanced intermediate L4. The subsequent deprotection of Boc group followed by either thiosuccinylation or thiomyristoylation [21] of the ε-amino group led to the production of probe 2B or 2C, respectively. Good to excellent yields were obtained for the above-mentioned reactions. All the new compounds were fully characterized by ^1^H, ^13^C NMR, and HRMS.

Inhibition of sirtuin deacetylase activity by these ABPs was examined using the HPLC assay as described in “Materials and Methods”. It is important to point out that our current study does not include SIRT4 and SIRT7, which are difficult to be expressed in *E. coli* cells. Probe 2A, with a thioacetyl warhead, demonstrated broad inhibition against SIRT1, SIRT2, SIRT3, and SIRT6 with IC_50_s in the low micromolar range (Table 1). It exhibited 3-fold increased inhibition of SIRT1 and 2-fold increased inhibition of SIRT3 compared with probe 1A, its benzophenone counterpart. More strikingly, probe 2A also showed mild inhibition against SIRT6. The value of the ABPs is that they may target a family of catalytically related enzymes and provide a quantitative readout of the functional status of individual family members simultaneously [38]. Replacing the sterically hindered benzophenone with diazirine indeed improved the inhibitory effects. A similar pattern was observed for probe 2C, which had an expanded target pool relative to probe 1C. It inhibited SIRT2 with an IC_50_ of 7.4 ± 1.3 μM, a 3-fold increase compared with 1C, and a nearly 2-fold selectivity over SIRT6. SIRT1 can be inhibited by probe 2C as well with an IC_50_ of 79.9 ± 7.6 μM. A previous study suggested that SIRT1, SIRT2, and SIRT6 have strong deacylase activity against long-chain fatty acyl groups such as myristoyl [39]. Not surprisingly, the thiomyristoyl probe 2C was an inhibitor of these three sirtuin isoforms. Probe 2B was a potent SIRT5-selective inhibitor with an IC_50_ of 1.9 ± 0.6 μM, which is a nearly 1.7-fold increase compared with 1B. Structural studies suggest that SIRT5 has a large binding pocket with critical Y102 and R105 residues securing negatively charged acyl substrates such as succinyl and malonyl lysines [40]. This unique feature renders thiosuccinyllysine peptide such as probe 2B selective SIRT5 inhibitor.

### 2.2. Diazirine-Containing Probes Demonstrate Improved Labeling Efficiencies

**Figure 3:**
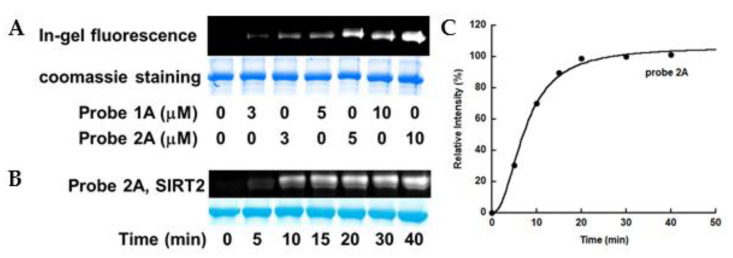
Comparison of labeling efficiencies of probes 1A and 2A. (**A**) Concentration-dependent labeling of SIRT2. SIRT2 (10 μM) was incubated with 500 μM NAD^+^ and indicated concentrations of probe 1A or 2A in 100 mM phosphate buffer pH 7.5. The samples were incubated at 37 °C for 10 min, subjected to UV irradiation for 1 h, and then “click” conjugated to TAMRA-azide as described in “Materials and Methods”; (**B**) and (**C**) Time-dependent labeling of SIRT2 by probe 2A. The labeling reached plateau within 20 min.

The improved inhibitory effects of the 2nd generation ABPs prompted us to evaluate the labeling efficiency of these new probes. We first compared the abilities of benzophenone-based probe 1A and diazirine-based probe 2A to label recombinant SIRT2. The enzyme was incubated with NAD^+^ and the probe, and then irradiated at 365 nm for 1 h. Subsequently, the sample was conjugated to TAMRA-azide via Cu(I)-catalyzed “click” reaction. The sample was resolved by SDS-PAGE and detected by in-gel fluorescence scanning. Probe 2A labeled SIRT2 more intensively than probe 1A at all the concentrations examined (Figure 3A), suggesting a higher labeling efficiency of the diazirine-containing probe 2A. Probe 2A labeled SIRT1, SIRT2, SIRT3, and SIRT6 to different extents in dose-dependent manners (Figure S1).

Time dependence of the labeling mediated by probes 1A and 2A was also examined. The labeling of SIRT2 by probe 2A reached saturation within 20 min (Figure 3B,C), while the labeling by probe 1A reached plateau at round 60 min [21]. For all the subsequent experiments using 2nd generation probes, 20 min irradiation time was used.

**Figure 4:**
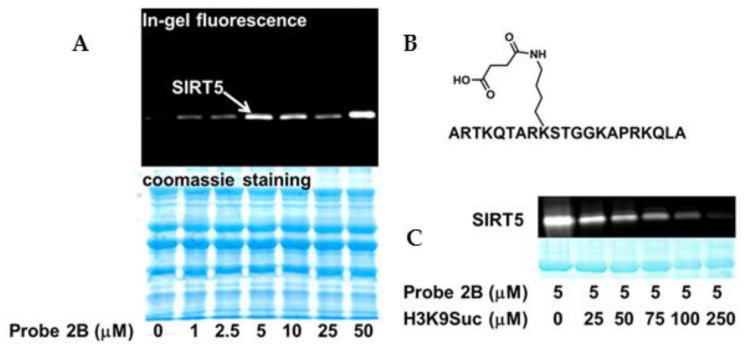
Labeling of SIRT5 by probe 2B. (**A**) Dose-dependent labeling of SIRT5 by probe 2B in recombinantly expressed, partially purified sirtuin mixture. SIRT1, SIRT2, SIRT3, SIRT5, and SIRT6 were mixed and incubated with 500 μM NAD^+^ and increasing concentrations of 2B. The samples were incubated at 37 °C for 10 min and subjected to UV irradiation for 20 min, and then “click” conjugated to TAMRA-azide; (**B**) Chemical structure of H3K9Suc; (**C**) Peptide competition. The labeling of SIRT5 by probe 2B decreased with increasing concentrations of the competitor peptide, H3K9Suc.

Probe 2B, being a selective SIRT5 inhibitor, was also selective for SIRT5 in the labeling experiments. It showed robust labeling of SIRT5 in a concentration-dependent fashion but negligible labeling of the other sirtuin isoforms in a partially purified recombinant sirtuin mixture (Figure 4A). The on-target effect of probe 2B was further confirmed by the peptide competition analysis. H3K9Suc (Figure 4B), a synthetic peptide that is identical to the N-terminus of histone H3 bearing a succinylated lysine 9 mark, is a known SIRT5 substrate [21]. H3K9Suc was titrated into SIRT5 in the presence of probe 2B. The labeling of SIRT5 by probe 2A was outcompeted by H3K9Suc in a dose-dependent manner, which is consistent with the notion that thioacyllysine mimics the action of acyllysine to target sirtuin active site.

**Figure 5:**
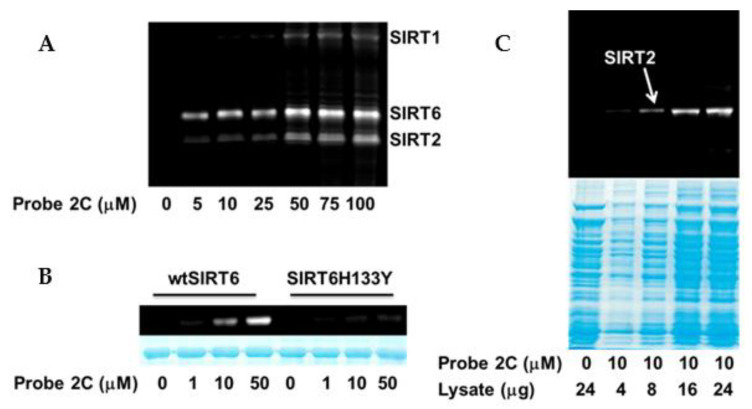
Activity-based labeling by probe 2C. (**A**) Labeling of SIRT1, SIRT2, and SIRT6 in sirtuin mixtures; (**B**) Probe 2C demonstrated dose-dependent labeling of SIRT6, but failed to label SIRT6H133Y, a catalytically inactive mutant; (**C**) Probe 2C selectively labeled SIRT2 in SIRT2-overexpressing HEK293 cell lysate.

Benzophenone-containing probe 1C selectively labeled SIRT6 in a complex protein mixture [21]. Its diazirine counterpart, probe 2C, outperformed it by highlighting SIRT1, SIRT2, and SIRT6 in the sirtuin mixture (Figure 5A), which is consistent with the initial inhibition analysis (Table 1). The broader target range of probe 2C (Figure S2) carrying the thiomyristoyllysine warhead is expected because a previous study indicated that several human sirtuins harbor intrinsic defatty-acylase activity including SIRT1, SIRT2, and SIRT6 [39]. This probe was activity-based as it demonstrated concentration-dependent labeling of wild-type SIRT6 (wtSIRT6) but failed to label the catalytically inactive mutant SIRT6H133Y to any appreciable levels (Figure 5B). Remarkably, probe 2C was also able to label individual sirtuin isoforms in cell lysates. HEK293 cells overexpressing human SIRT2 were lysed, and the lysate was subjected to the labeling by 10 μM probe 2C. A clean and selective labeling of SIRT2 was detected (Figure 5C). The labeling was even observed in the sample with merely 4 μg cell lysate, demonstrating the superb sensitivity of the probe.

Taken together, our results suggested that the 2nd generation probes with diazirine photoaffinity tag required shorter irradiation time to achieve similar or higher labeling efficiency compared to the 1st generation benzophenone-containing probes. Several probes also demonstrated broader labeling targets, reduced non-specific labeling as well as high sensitivity.

### 2.3. Diazirine-Containing ABPs Are Cell Permeable Sirtuin Inhibitors

**Figure 6:**
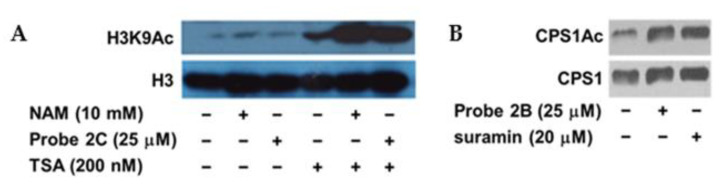
Probes 2B and 2C are cell permeable sirtuin inhibitors. (**A**) Western blot showing increased acetylation level of H3K9 (H3K9Ac) in HEK293 cells treated with probe 2C; (**B**) Incubation with probe 2B or suramin increased CPS1Ac level in HEK293 cells.

The ABPs structurally mimic acylated lysine substrates. However, the presence of substituents could potentially alter their cell permeability, cellular distribution, and target engagement ability. It is essential to determine if these probes are cell permeable and capable of inhibiting endogenous sirtuin activity. The sirtuin family of proteins are often referred to as the class III histone deacetylases (HDACs). Unlike class I and class II HDACs, which are Zn^2+^-dependent, sirtuins are NAD^+^-dependent. Our previous study indicated that trichostatin A (TSA), a class I/II HDAC inhibitor, and probe 1A acted coordinately to enhance the acetylation level of p53 lysine 382 [21]. Similarly, other sirtuins may not be the only deacetylase of their endogenous substrates. Therefore, the inhibitory effect of the other ABPs may only become apparent when class I/II HADCs are inhibited at the same time. HEK293 cells were cultured in the presence of probe 2C and/or other HDAC inhibitors. Subsequently, the cell lysates were collected and probed for acetylated H3K9 (H3K9Ac), which is an endogenous substrate of SIRT6. Treatment with nicotinamide (NAM, a physiological sirtuin inhibitor) or probe 2C alone did not cause any appreciable changes (Figure 6A). Incubation with TSA increased the acetylation level of H3K9. The combination treatments, either TSA and NAM or TSA and probe 2C, resulted in a significantly elevated level of H3K9Ac, suggesting that probe 2C is a cell permeable SIRT6 inhibitor.

The cellular activity of probes 2A and 2B was also investigated. 2A, very similar to 1A [21], was able to increase the acetylation level of p53 lysine 382, an endogenous target of SIRT1, when combined with TSA (Figure S3). SIRT5 is known to deacetylate and activate carbamoyl phosphate synthetase I (CPS1), which is the enzyme catalyzing the first step of the urea cycle [41]. Cells were treated with either probe 2B or a known SIRT5 inhibitor, suramin, before the acetylation level of CPS1 (CPS1Ac) was examined. Both probe 2B and suramin increased CPS1Ac level compared to the control sample (Figure 6B), which is presumably through the inhibition of SIRT5.

**Figure 7:**
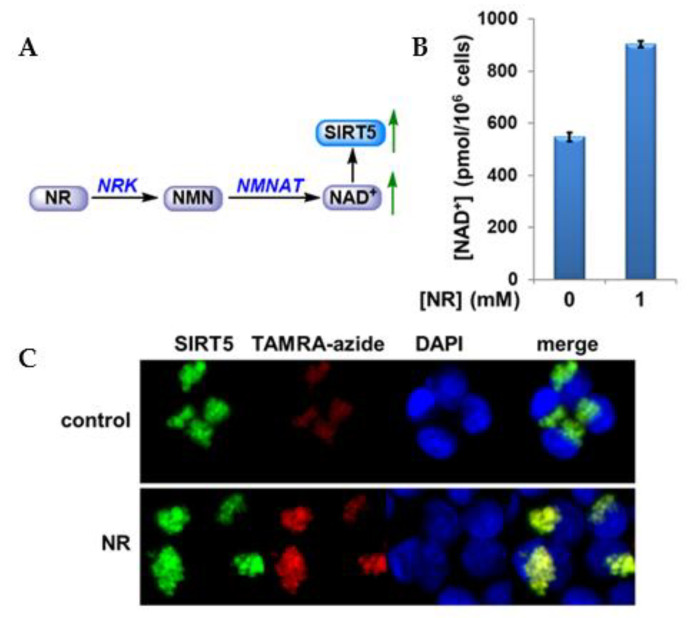
In-cell labeling of SIRT5 with probe 2B. (**A**). Nicotinamide riboside (NR) pathway; (**B**). Cellular NAD^+^ concentrations; (**C**) HEK293 cells overexpressing SIRT5 and treated with NR showed increased labeling intensity compared with control cells.

Encouraged by our initial cellular studies, we continued our effort on evaluating the dynamic enzyme activity change under different cellular conditions using ABPs in combination with imaging approach. Results from our lab and others have indicated that sirtuins have evolved to respond to the availability of NAD^+^, which is an essential currency of cellular metabolism and DNA repair, and convert this information to many different biological outputs [21,22,41,42,43]. HEK293 cells overexpressing SIRT5 were subjected to a treatment known to increase the cellular NAD^+^ level. Nicotinamide riboside (NR), a trace nutrient found in milk, has been identified as an efficient NAD^+^ boosting agent [44,45,46,47,48]. NR can be converted to nicotinamide mononucleotide (NMN) by NR kinases (NRK1 and NRK2). Then, NMN can be transformed to NAD^+^ by the action of NMN adenylyltransferase (NMNAT) (Figure 7A). Indeed, incubation with 1 mM NR for 6 h dramatically increased cellular NAD^+^ concentration by 170% as determined by NAD^+^ cycling assay (Figure 7B). Then, the cells were incubated with 50 μM probe 2B and irradiated at 365 nm for 15 min on ice. Subsequently, the cells were treated with freshly prepared “click” staining mix as described in “Materials and Methods”. After the “click” reaction, cells were incubated with anti-SIRT5 antibody before the images were acquired. NAD^+^ elevation led to increased labeling of SIRT5 (Figure 7C, TAMRA-azide) without changing the SIRT5 protein level (Figure 7C, SIRT5). Repletion of intracellular NAD^+^ pool with biosynthetic precursors such as NR has been suggested as new therapeutic avenues for lifespan and healthspan extension [49,50,51,52], and many of the beneficial effects of NAD^+^ restoration seem to be mediated by sirtuin activities. This is why sirtuins sometimes are referred to as the NAD^+^ sensors. The remarkably simple ABP engineered in our lab showed promising results for SIRT5 profiling in response to cellular NAD^+^ perturbations. It provided direct evidence of the regulatory link between energy homeostasis and sirtuin functions.

## 3. Materials and Methods

### 3.1. Reagents and Instruments

All reagents were purchased from Sigma-Aldrich (St. Louis, MO, USA) or Fisher Scientific (Pittsburgh, PA, USA) and were of the highest purity commercially available. HPLC was performed on a Dionex Ultimate 3000 HPLC (Thermo Electron, Madison, WI, USA) system equipped with a diode array detector using Macherey-Nagel C18 reverse-phase column (Macherey-Nagel, Bethlehem, PA, USA). NMR spectra were acquired on a Bruker AVANCE III 500 MHz high-field NMR spectrometer (Bruker, Billerica, MA, USA) and the data were processed using Topspin software. Radiolabeled samples were counted in a Beckman LS6500 scintillation counter (Beckman, Indianapolis, IN, USA). HRMS spectra were acquired with either a Waters Micromass Q-tof Ultima (Waters, Milford, MA, USA) or a Thermo Scientific Q-Exactive hybrid Quadrupole Orbitrap (Thermo Scientific, San Jose, CA, USA). Fluorescence scanning was performed on a Biorad ChemiDoc MP (Biorad, Hercules, CA, USA) imaging system.

### 3.2. Synthetic Peptides

Synthetic peptides H3K9Ac: ARTKQTAR(K-Ac)STGGKAPRKQLAS, p53K382Ac: KKGQSTSRHK(K-Ac)LMFKTEG, and H3K9Suc: ARTKQTAR(K-Suc)STGGKAPRKQLA were synthesized and purified by Genscript. The peptides were purified by HPLC to a purity > 95%.

### 3.3. Protein Expression and Purification

Plasmids of SIRT1 (full length), SIRT2 (38-356), SIRT3 (102-399), and SIRT5 (34-302) were generous gifts from Dr. Hening Lin (Cornell University, Ithaca, NY, USA). SIRT6 was a gift from Cheryl Arrowsmith (Addgene plasmid #28271). The proteins were expressed and purified according to previously published protocols [22,53]. The identity of the protein was confirmed by tryptic digestion followed by LC-MS/MS analysis performed at the Vermont Genetic Network (VGN) Proteomics Facility. Protein concentrations were determined by Bradford assay (Thermo Fisher Scientific, Rockford, IL, USA).

### 3.4. Sirtuin Inhibition Assay

A typical reaction contained 500 μM NAD^+^ and 500 μM peptide substrate (H3K9Ac for SIRT2, SIRT3 and SIRT6, p53K382Ac for SIRT1 and SIRT5) of varying concentrations of a small molecule probe in 100 mM phosphate buffer pH 7.5. The reactions were initiated by the addition of 10 μM of sirtuin and were incubated at 37 °C before being quenched by 8 μL of 10% TFA. The incubation time was controlled so that the conversion of substrate was less than 15%. The samples were then injected on an HPLC fitted to a Macherey-Nagel Nucleosil C18 column. NAD^+^, NAM and AADPR (acetyl-ADP-ribose) peaks were resolved using a gradient of 0 to 20% methanol in 20 mM ammonium acetate. Chromatograms were analyzed at 260 nm. Reactions were quantified by integrating areas of peaks corresponding to NAD^+^ and AADPR. Rates were plotted as a function of small molecule probe concentration, and points were fitted to the following equation:

ν(%) = ν_0_(%) − [ν_0_(%)(10^x^)/(10^x^ + IC_50_)] (1)

where ν(%) represents the turnover rate expressed as percent enzymatic activity remaining, and ν_0_(%) represents the uninhibited turnover rate expressed as an enzymatic activity of 100%. The variable x represents the log[probe] in nanomolar. IC_50_ values were derived from this equation.

### 3.5. Labeling of Recombinant Sirtuin

A typical labeling experiment was performed as follows: in a 0.7 mL Eppendorf tube, purified recombinant human sirtuin (10 μM) was incubated with NAD^+^ (500 μM) and activity-based probe at 37 °C for 10 min. The sample was transferred to a clear-bottom 96-well plate, placed on ice, and irradiated at 365 nm with a UV-lamp in a cold room. Subsequently, ingredients of the “click-chemistry” including azide-fluor 545, CuSO_4_, reducing agent (tris(2-carboxyethyl)phosphine, TCEP) and stabilizing agent (tris[(1-benzyl-1H-1,2,3-triazol-4-yl)methyl]amine, TBTA) were added, and the sample was gently agitated at 250 rpm on a microshaker at room temperature for 30 min. Then, the sample was resolved by SDS-PAGE. To reduce the signal to noise ratio, the gel was destained to eliminate non-specific binding of free dyes. This was done in a mixture of methanol/distilled water/acetic acid (*v/v/v* = 4/5/1) at ambient temperature for 4 h. Then, destained gel was analyzed with in-gel fluorescence scanning using a Biorad ChemiDoc MP imager (excitation 532 nm, 580 nm cut-off filter and 30 nm band-pass). Finally, Coomassie blue staining was applied to provide loading control.

### 3.6. Cell Culture and Transfection

HEK293 cells were cultured in DMEM supplemented with 10% fetal bovine serum (FBS), 100 U/mL penicillin and 100 mg/mL streptomycin. Cells were maintained in a humidified 37 °C incubator with 5% CO_2_. Cells were transfected using lipofectamine 2000 (Invitrogen, Carlsbad, CA, USA) according to the manufacturer’s instructions.

### 3.7. Cell Lysate Labeling

Cells were harvested and lysed with RIPA buffer (Thermo Fisher Scientific, Rockford, IL, USA) supplemented with protease inhibitor cocktail (Thermo Fisher Scientific, Rockford, IL, USA). Protein concentration was determined by Bradford assay. A typical labeling experiment contained 4 to 24 μg of protein, 500 μM NAD^+^, and 10 μM probe in 100 mM phosphate buffer pH 7.5. The photoaffinity labeling, “click” conjugation to fluorescent dye and visualization were similar to the protocols for labeling recombinant sirtuins as described above.

### 3.8. Western Blot

The cell lysate was resolved on a 10% SDS-PAGE gel and transferred to Immobilon PVDF transfer membrane (Millipore, Burlington, MA, USA). The blot was blocked with 5% nonfat milk, probed with primary antibody targeting H3K9Ac (Sigma-Aldrich, St. Louis, MO, USA), H3 (Abcam, Cambridge, MA, USA), acetyllysine or CPS1 (Cell Signaling Technology, Beverly, MA, USA), washed with PBST, followed by incubation with anti-rabbit or anti-mouse HRP conjugated secondary antibody. Then, the signal was detected by SuperSignal West Pico Chemiluminescent substrate (Thermo Fisher Scientific, Rockford, IL, USA).

### 3.9. NAD^+^ Measurement

To the cell pellet was added 30 μL of ice-cold 7% perchloric acid; then, the sample was vortexed for 30 s and then sonicated on ice for 5 min. The vortex–sonication cycle was repeated three times. Then, the sample was centrifuged for 3 min at room temperature. Clear supernatant was taken out and neutralized to pH 7 with 3 M NaOH and 1 M phosphate buffer (pH 9). NAD^+^ level was then measured using NAD^+^ cycling assay as described previously [14].

### 3.10. Cellular Imaging

Cells were seeded in 35 mm glass-bottom dishes (MatTek, Ashland, MA, USA) and grew until 70 to 80% confluence. Then, the cells were incubated in the presence or absence of 1 mM NR for 6 h before the medium was discarded. The cells were gently rinsed with fresh growth medium three times. Subsequently, the cells were incubated with 50 μM probe 2B for 1 h before the culture dishes were placed on ice. The cells were irradiated at 365 nm in cold room for 15 min followed by fixation with 4% paraformaldehyde and permeabilization with 0.2% Triton X-100 in PBS. Then, the cells were treated with freshly prepared “click” staining mix (10 μM TAMRA-azide, 1 mM CuSO_4_ and 1 mM TBTA in 2 mL of deionized water), followed by the addition of 2 mM TCEP. The incubation lasted for 30 min at room temperature in the dark with gentle agitation. The cells were rinsed with PBS three times. After the “click” reaction, cells were incubated with anti-SIRT5 antibody (Cell Signaling Technology, Beverly, MA, USA) at room temperature for 1 h, washed three times with 0.05% Tween-20 in PBS, and then incubated with anti-rabbit Alexa Fluor^®^488 IgG antibody (Cell Signaling Technology, Beverly, MA, USA) for 30 min at room temperature. Cells were rinsed again with 0.05% Tween-20 in PBS three times with gentle agitation. For nuclear DNA staining, the cells were incubated with 3 μM DAPI in PBS for 10 min at room temperature. Cells were rinsed with PBS three times and with deionized water once before images were acquired.

Fluorescence microscopy was carried out on a Leica TCS SP5 confocal microscope (Leica Microsystems, Mannheim, Germany). Alexa Fluor 488 was excited at 488 nm, and emission was obtained at 495–525 nm. TAMRA-azide was excited at 543 nm, and fluorescent emission was obtained at 565–595 nm. Images were acquired and processed using Leica Application Suite Advanced Fluorescence (LAS AF) software.

### 3.11. Synthesis

#### 3.11.1. Synthesis of L1

L1 was prepared as described previously [36,37]. ^1^H NMR (CDCl_3_, 400 MHz) δ (ppm): 1.29 (m, 2H), 1.49 (m, 2H), 1.69 (t, *J* = 7.8 Hz, 2H), 1.94 (t, *J* = 2.6 Hz, 1H), 2.05 (t, *J* = 7.5 Hz, 2H), 2.13 (m, 2H). ^13^C NMR (CDCl_3_, 100 MHz) δ (ppm): 17.83, 22.60, 27.96, 28.30, 29.40, 31.51, 69.11, 83.30, 178.16. HRMS (*m*/*z*): calculated for C_9_H_13_N_2_O_2_ (M + H): 181.0972; found: 181.0976.

#### 3.11.2. Synthesis of L2

L2 was prepared as reported previously [21]. ^1^H NMR (CD_3_OD, 300 MHz) δ (ppm): 1.37 (d, *J* = 4.4 Hz, 3H), 1.39 (d, *J* = 4.2 Hz, 3H), 1.57 (m, 2H), 1.69 (m, 2H), 1.88 (m, 2H), 2.43 (s, 3H), 3.59 (t, *J* = 7.0 Hz, 2 H), 3.70 (s, 3H), 4.39 (m, 2H), 4.55 (m, 1H).

#### 3.11.3. Synthesis of Probe 2A

To a solution of intermediate L2 (121 mg, 0.336 mmol) in 3 mL of THF was added a solution of intermediate L1 (50 mg, 0.28 mmol) in 2 mL of THF. PyBOP (210 mg, 0.403 mmol) and DIPEA (104 mg, 0.806 mmol) were introduced into the reaction mixture. The reaction was stirred at room temperature until completion. Reaction was quenched with water. Aqueous layer was extracted with ethyl acetate. Combined organic layer was washed with brine and dried over Na_2_SO_4_. Solvent was evaporated under reduced pressure, and the crude product was purified by silica gel column chromatography (ethyl acetate with 3% methanol) to afford probe 2A (124 mg, 0.24 mmol, 85%) as a pale yellow oil^1^H NMR (CDCl_3_, 500 MHz) δ (ppm): 1.31 (d, *J* = 7.2 Hz, 3H), 1.34 (d, *J* = 7.2 Hz, 3H), 1.42 (m, 2H), 1.51 (m, 2H), 1.59 (m, 2H), 1.84 (m, 4H), 1.93 (t, *J* = 2.6 Hz, 1H), 2.14 (stack, 6H), 2.47 (s, 3H), 3.53 (m, 2H), 3.68 (s, 3H), 4.41 (m, 1H), 4.48 (m, 1H), 4.71 (m, 1H). ^13^C NMR (CD_3_OD, 125 MHz) δ (ppm): 17.90, 18.24, 19.15, 20.74, 21.82, 25.53, 29.84, 33.58, 35.01, 45.98, 46.24, 46.27, 48.25, 49.14, 52.52, 52.93, 67.55, 71.50, 85.99, 171.98, 172.30, 172.99, 175.31, 200.81. HRMS (*m*/*z*): calculated for C_24_H_39_N_6_O_5_S (M + H): 523.2697; found: 523.2701.

#### 3.11.4. Synthesis of L3

L3 was prepared as described previously [21]. ^1^H NMR (CDCl_3_, 500 MHz) δ (ppm): 1.35 (d, *J* = 8.9 Hz, 3H), 1.37 (d, *J* = 8.7 Hz, 3H), 1.40 (s, 9H), 1.51 (m, 4H), 1.65 (m, 2H), 1.80 (m, 1H), 2.55 (m, 2H), 3.07 (m, 2H), 3.38 (m, 1H), 3.71 (s, 3H), 4.48 (m, 2H), 4.68 (m, 1H).

#### 3.11.5. Synthesis of L4

To a solution of intermediate L3 (150 mg, 0.37 mmol) in 5 mL of THF was added a solution of intermediate L1 (67 mg, 0.37 mmol) in 3 mL of THF. PyBOP (232 mg, 0.45 mmol) and DIPEA (116 mg, 0.9 mmol) were introduced into the reaction mixture. The reaction was stirred at room temperature until completion. The reaction was quenched with water. Aqueous layer was extracted with ethyl acetate. Combined organic layer was washed with brine and dried over Na_2_SO_4._ Solvent was evaporated under reduced pressure, and the crude product was purified by silica gel column chromatography (CH_2_Cl_2_ with 10–20% methanol) to afford L4 (168 mg, 0.30 mmol, 81%) as a pale yellow oil. ^1^H NMR (CDCl_3_, 500 MHz) δ (ppm): 1.30 (m, 2H), 1.34 (m, 4H), 1.38 (s, 9H), 1.41 (d, *J* = 7.5 Hz, 6H), 1.46 (m, 2H), 1.61 (m, 1H), 1.76 (m, 3H), 1.91 (t, *J* = 2.7 Hz, 1H), 2.02 (m, 2H), 2.11 (m, 2H), 3.01 (m, 2H), 3.73 (s, 3H), 4.57 (m, 1H), 4.68 (m, 1H), 4.81 (m, 2H). ^13^C NMR (CDCl_3_, 125 MHz) δ (ppm): 18.09, 18.53, 19.15, 21.82, 22.59, 25.53, 26.68, 28.36, 29.45, 29.84, 32.47, 35.02, 48.05, 48.92, 52.43, 53.42, 66.92, 67.53, 71.48, 79.08, 156.21, 162.87, 166.74, 171.78, 173.14. HRMS (*m*/*z*): calculated for C_27_H_45_N_6_O_7_ (M + H): 565.3344; found: 565.3342.

#### 3.11.6. Synthesis of L5

To a solution of intermediate L4 (67 mg, 0.12 mmol) in 1 mL of anhydrous CH_2_Cl_2_ was added TFA (1 mL) and triethylsilane (0.1 mL). The reaction was allowed to proceed for 45 min before solvent was evaporated under reduced pressure to afford L5 (55 mg, 0.12 mmol, quantitative) as a yellow oil. The crude product was used for the next step without further purification. ^1^H NMR (CDCl_3_, 500 MHz) δ (ppm): 1.35 (d, *J* = 7.2 Hz, 3H), 1.38 (d, *J* = 6.9 Hz, 3H), 1.38 (m, 2H), 1.65 (m, 3H), 1.73 (m, 5H), 1.86 (m, 2H), 1.94 (t, *J* = 2.7 Hz, 1H), 2.18 (m, 2H), 2.44 (m, 2H), 3.61 (m, 2H), 3.71 (s, 3H), 4.52 (m, 2H), 4.78 (m, 1H). ^13^C NMR (CD_3_OD, 125 MHz) δ (ppm): 17.50, 18.14, 18.67, 24.11, 24.61, 28.48, 29.74, 32.11, 32.80, 32.85, 33.25, 47.03, 50.32, 52.89, 55.40, 70.27, 84.22, 169.85, 173.06, 174.42, 174.69, 174.87. HRMS (*m*/*z*): calculated for C_22_H_37_N_6_O_5_ (M + H): 465.2820; found: 465.2826.

#### 3.11.7. Synthesis of Probe 2B

To a solution of L5 (56 mg, 0.12 mmol) in 1 mL of methanol was added 1 mL of 5% Na_2_CO_3_ at 0 °C dropwise. The mixture was stirred at 0 °C for 30 min before a solution of *tert*-butyl thiophenyl mono-thiosuccinate (51 mg, 0.18 mmol) in methanol was added. The reaction was allowed to warm to room temperature and stir until completion. The reaction mixture was acidified by 1 N HCl to pH 2. Aqueous layer was extracted with ethyl acetate three times. Combined organic layer was washed with brine and dried over Na_2_SO_4_. Solvent was removed under reduced pressure, and the crude product was purified by silica gel column chromatography (CH_2_Cl_2_ with 10~20% methanol) to afford probe 2B (58 mg, 0.10 mmol, 83%) as a yellow viscous oil ^1^H NMR (CDCl_3_, 500 MHz) δ (ppm): 1.29 (m, 2H), 1.34 (d, *J* = 6.6 Hz, 3H), 1.37 (d, *J* = 7.2 Hz, 3H), 1.38 (m, 2H), 1.48 (m, 2H), 1.60 (m, 2H), 1.67 (m, 2H), 1.79 (m, 2H), 1.92 (t, *J* = 2.6 Hz, 1H), 1.98 (m, 2H), 3.12 (m, 2H), 2.68 (m, 1H), 2.77 (m, 1H), 2.88 (m, 2H), 3.47 (m, 1H), 3.66 (m, 1H), 3.73 (s, 3H), 4.56 (m, 2H), 4.63 (m, 1H). ^13^C NMR (CD_3_OD, 100 MHz) δ (ppm): 17.53, 18.19, 19.89, 23.04, 24.61, 26.90, 27.98, 28.49, 31.20, 32.86, 33.28, 36.29, 47.04, 50.34, 52.89, 55.50, 68.40, 69.02, 72.71, 87.29, 164.78, 169.55, 174.16, 174.68, 174.84, 196.73. HRMS (*m*/*z*): calculated for C_26_H_41_N_6_O_7_S (M + H): 581.2752; found: 581.2754.

#### 3.11.8. Synthesis of Probe 2C

To a solution of L5 (77 mg, 0.17 mmol) in 2 mL of methanol was added 2 mL of 5% Na_2_CO_3_ at 0 °C dropwise. The mixture was stirred at 0 °C for 30 min before a solution of thiophenyl thiomyristate (86 mg, 0.26 mmol) in methanol was added. The reaction was allowed to warm to room temperature and stir until completion. The reaction mixture was acidified by 1 N HCl to pH 2. Aqueous layer was extracted with ethyl acetate three times. The combined organic layer was washed with brine and dried over Na_2_SO_4_. Solvent was removed under reduced pressure, and the crude product was purified by silica gel column chromatography (CH_2_Cl_2_ with 5% methanol) to afford probe 2C (107 mg, 0.15 mmol, 91%) as a yellow oil. ^1^H NMR (CDCl_3_/CD_3_OD, 400 MHz) δ (ppm): 0.84 (t, *J* = 6.3 Hz, 3H), 1.22 (stack, 20H), 1.35 (d, *J* = 6.8 Hz, 3H), 1.36 (d, *J* = 7.1 Hz, 3H), 1.43 (m, 2H), 1.52 (m, 2H), 1.66 (m, 4H), 1.80 (m, 3H), 1.88 (m, 1H), 2.01 (t, *J* = 2.3 Hz, 1H), 2.12 (m, 4H), 2.56 (t, *J* = 7.7 Hz, 2H), 3.56 (t, *J* = 6.8 Hz, 2H), 3.69 (s, 3H), 4.38 (m, 2H), 4.54 (m, 1H). ^13^C NMR (CDCl_3_, 125 MHz) δ (ppm): 14.05, 18.02, 18.53, 19.14, 21.81, 22.60, 22.73, 25.52, 26.67, 26.99, 29.03, 29.27, 29.39, 29.49, 29.58, 29.59, 29.61, 29.65, 29.83, 31.84, 35.00, 45.64, 46.70, 48.16, 49.05, 52.46, 66.88, 67.54, 71.47, 85.98, 166.85, 171.77, 172.10, 172.98, 194.63. HRMS (*m*/*z*): calculated for C_36_H_63_N_6_O_5_S (M + H): 691.4575; found: 691.4576.

## 4. Conclusions

The main focus of the current study was to engineer ABPs with photoactivatable diazirine group for the profiling of sirtuin activities in complex native environment. Indeed, the new generation of ABPs outperformed their benzophenone-based counterparts in terms of labeling efficiency, target protein range, and irradiation time. In turn, the shorter irradiation time resulted in reduced non-specific labeling, which was another benefit of the new probes. These group of ABPs labeled individual recombinant proteins, which were different isoforms in protein mixtures as well as the whole cell lysate. The cell permeability and bioactivity of the probes were further demonstrated in the cellular imaging experiment in which the active SIRT5 content change in response to elevated intracellular NAD^+^ level was captured via the labeling strategy. These new tools will provide an unparalleled platform to address sirtuin activity questions. They would substantially accelerate investigations on sirtuin biological functions and determine how sirtuins interact with cellular stimuli, such as hormones, calorie intake, and stresses as well as cell cycle changes.

## Figures and Tables

**Table 1:** IC_50_ values of activity-based chemical probes (ABPs).^1^

Sirtuin	Substrate	IC_50_ (μM)					
		1A	1B	1C	2A	2B	2C
SIRT1	p53K382Ac	39 ± 2.8	>5000	>1000	11.4 ± 2.7	>2000	79.9 ± 7.6
SIRT2	H3K9Ac	7 ± 0.5	>5000	21.8 ± 4.0	17.2 ± 3.3	>5000	7.4 ± 1.3
SIRT3	H3K9Ac	166 ± 23	>5000	>5000	77.9 ± 5.4	>5000	>2000
SIRT5	p53K382Ac	NI ^2^	3.2 ± 0.4	>5000	NI	1.9 ± 0.6	>5000
SIRT6	H3K9Ac	NI	>5000	7.8 ± 1.1	151.2 ± 21.9	>5000	13.7 ± 3.2

^1^ IC_50_ values were determined by HPLC assay as described in “Materials and Methods”. ^2^ Not inhibited.

## Supplementary Materials

The following are available online. Figure S1: Dose-dependent labeling of human sirtuins by probe 2A; Figure S2: Concentration-dependent labeling of sirtuins by probe 2C; Figure S3: Probe 2A is a cell permeable SIRT1 inhibitor. Figure S4: NMR spectra of the probes.
