# Supplementary material for: Development of Second Generation Activity-Based Chemical Probes for Sirtuins
Source: Molecules. 2020 Dec 22;26(1):11. doi: 10.3390/molecules26010011 (PMC7792806; doi:10.3390/molecules26010011)

## Supporting Information

# Development of Second Generation Activity-based Chemical Probes for Sirtuins

Alyson C. Curry [1], Elizabeth Barton [1], Wenjia Kang [1], Daniel V. Mongeluzi [1] and Yana Cen [1,2,*]

[1] Department of Medicinal Chemistry, Virginia Commonwealth University, Richmond, VA 23219;
[2] Institute for Structural Biology, Drug Discovery and Development, Virginia Commonwealth University, Richmond, VA 23219;

***** Correspondence: ceny2@vcu.edu; Tel.: +01-804-828-7405

**Figure S1.** Dose-dependent labeling of human sirtuins by probe 2A.

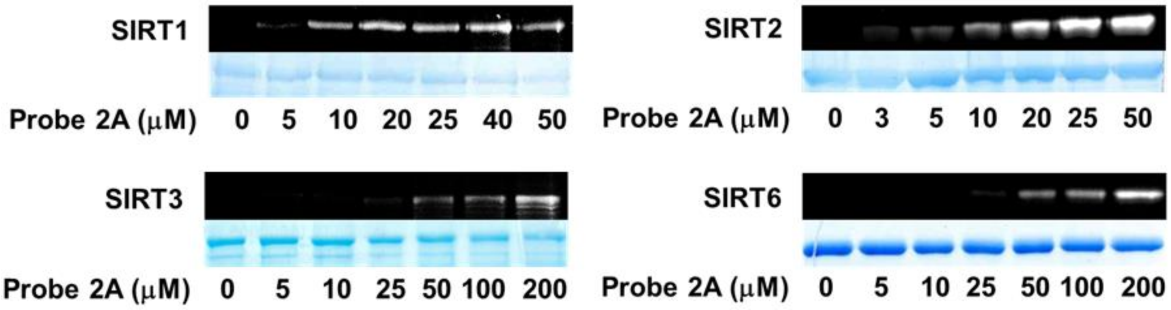

**Figure S2.** Concentration-dependent labeling of sirtuins by probe 2C.

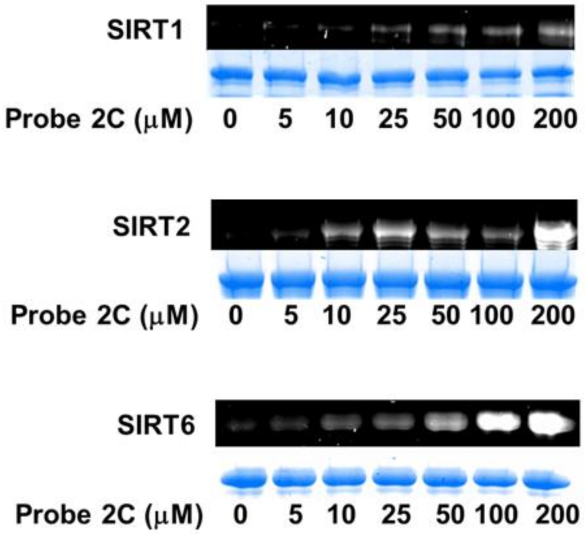

**Figure S3.** Probe 2A is a cell permeable SIRT1 inhibitor.

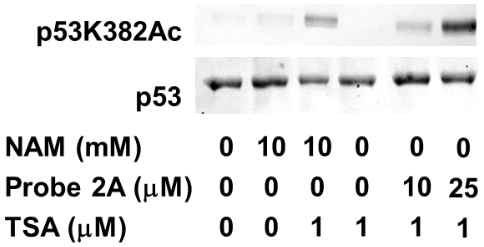

**Figure S4.** NMR spectra of the probes.

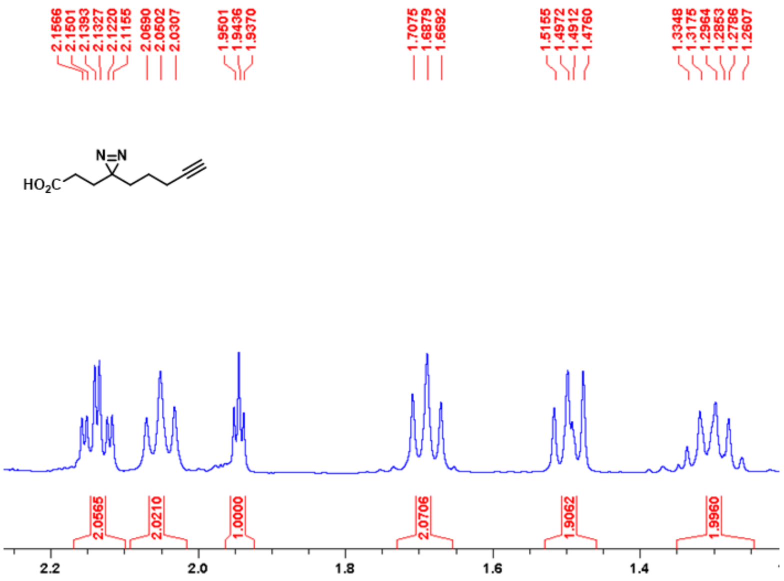

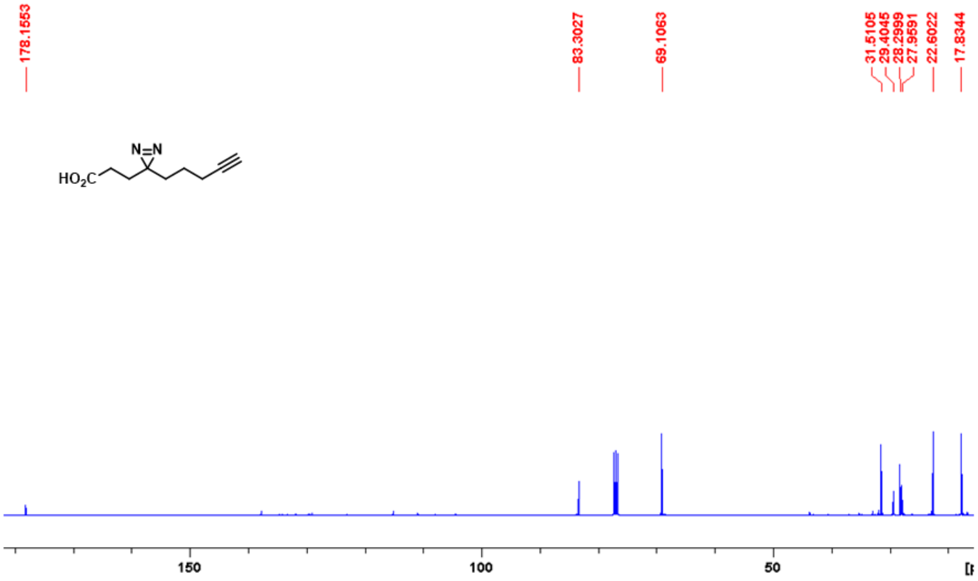

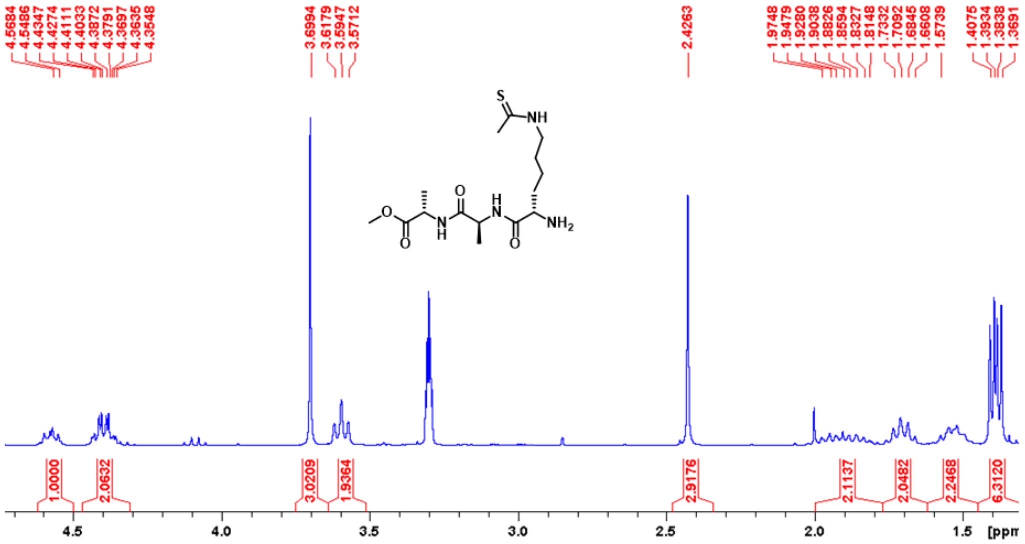

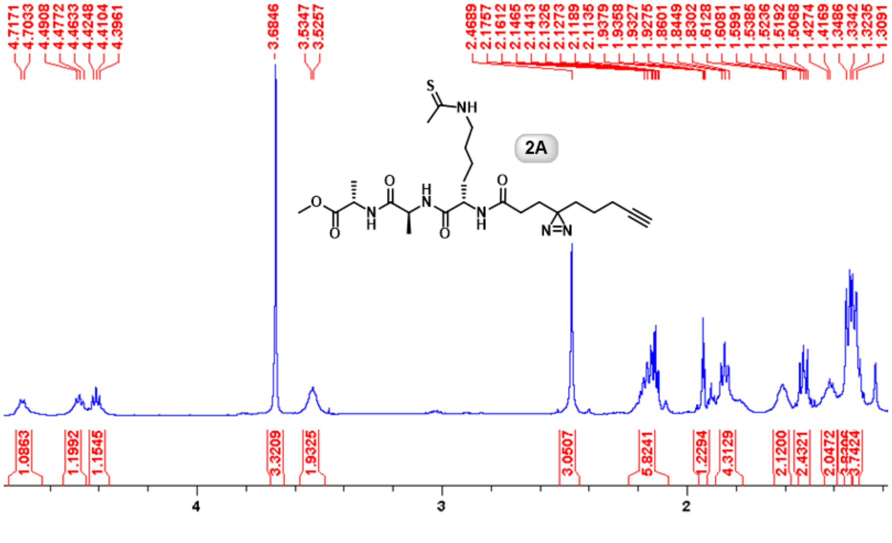
2A

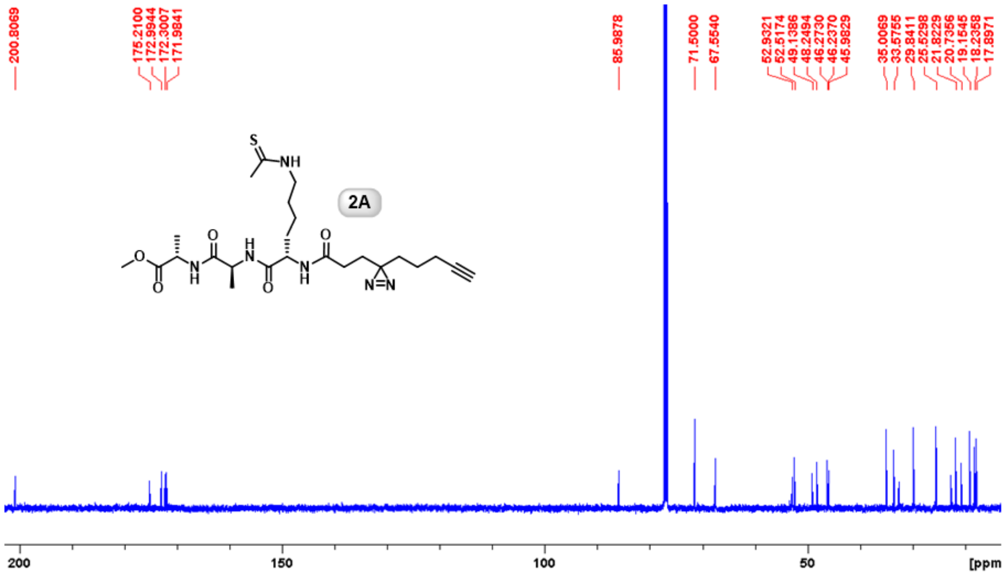

200.8069
175.2100
172.5944
172.3007
171.9841
85.9878
71.5000
67.5540
52.9321
52.5174
49.1386
48.2494
46.2730
46.2370
45.9829
35.0069
33.5765
29.8411
25.5298
21.8229
20.7356
19.1545
18.2358
17.8971
2A
200
150
100
50
[ppm


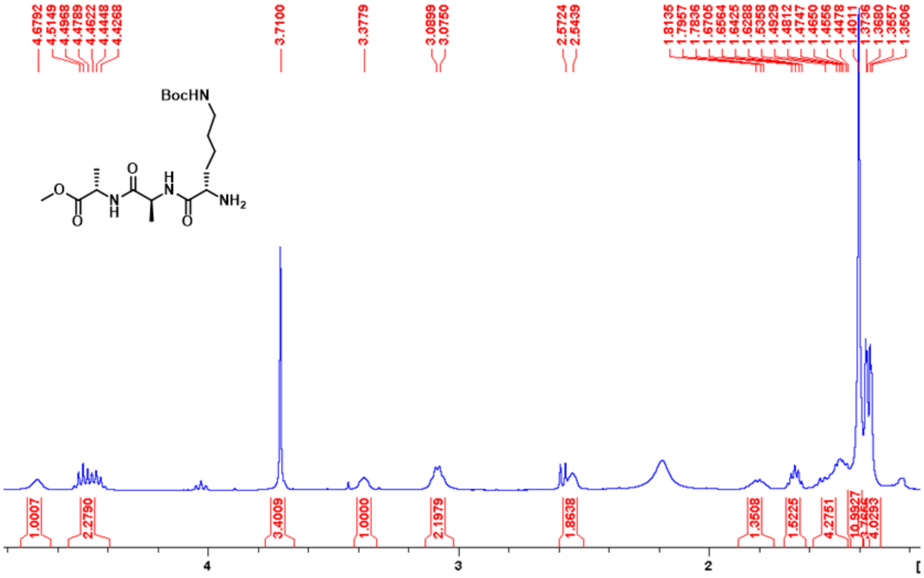

4.6792
4.5149
4.4968
4.4789
4.4622
4.4448
4.4268
3.7100
3.3779
3.0899
3.0750
2.5724
2.5439
1.8135
1.7957
1.7836
1.6705
1.6564
1.6425
1.6288
1.5358
1.4929
1.4812
1.4747
1.4650
1.4556
1.4478
1.4011
1.3736
1.3680
1.3557
1.3506
BocHN
NH2
1.0007
2.2790
3.4009
1.0000
2.1979
1.8638
1.3508
1.5225
4.2751
10.9927
4.0293
4
3
2

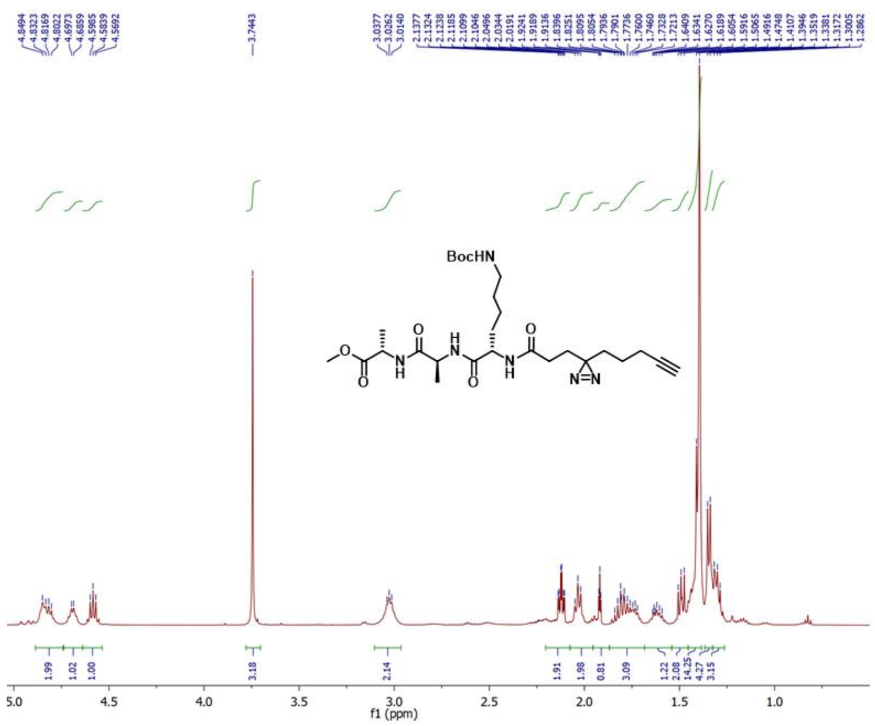

4.8494
4.8323
4.8169
4.8022
4.6973
4.6859
4.5985
4.5839
4.5692
3.7443
3.0377
3.0262
3.0140
BocHN
1.99
1.02
1.00
3.18
2.14
1.91
1.98
0.81
3.09
1.22
2.08
14.25
4.27
3.15
f1 (ppm)


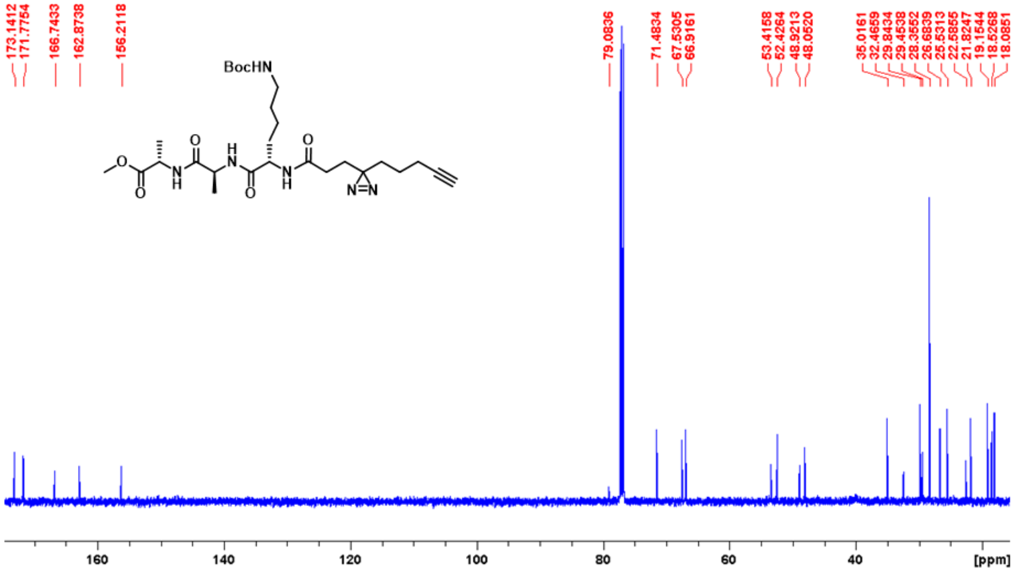

173.1412
171.7754
166.7433
162.8738
156.2118
79.0836
71.4834
67.5305
66.9161
53.4158
52.4264
48.9213
48.0520
35.0161
32.4659
29.8434
29.4538
28.3552
26.6839
25.5313
22.5855
21.8247
19.1544
18.5268
18.0851
BocHN
[ppm]

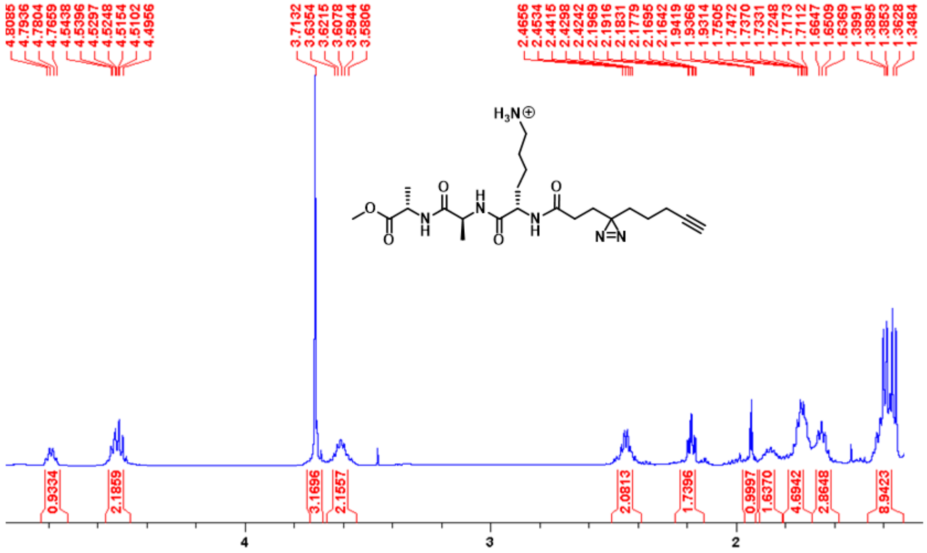
H3N⊕
O
H
N
O
N
H
O
N
H
O
N=N
4
3
2
0.9334
2.1859
3.1696
2.1557
2.0813
1.7396
0.9997
1.6370
4.6942
2.8648
8.9423

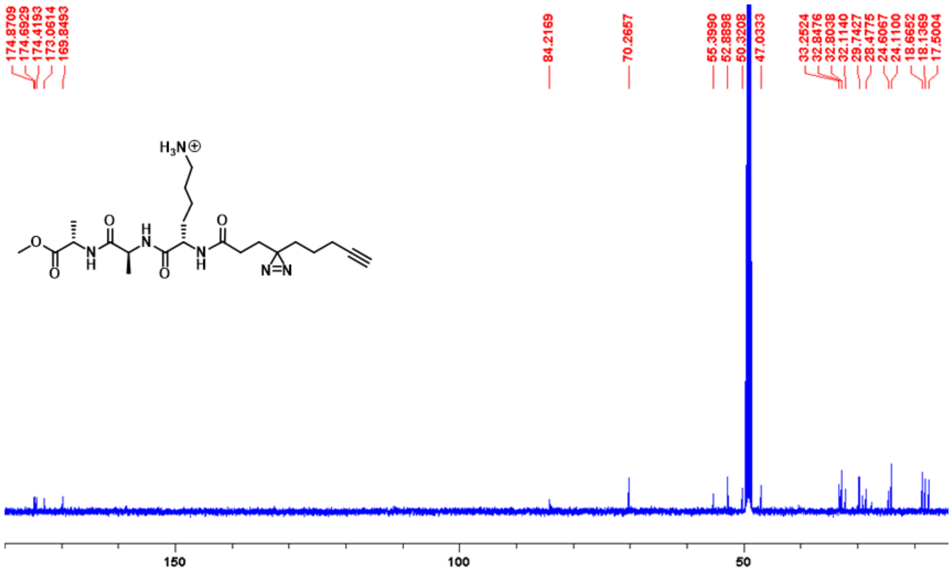
174.8709
174.6929
174.4193
173.0614
169.8493
84.2169
70.2657
55.3990
52.8898
50.3208
47.0333
33.2524
32.8476
32.8038
32.1140
29.7427
28.4775
24.6067
24.1100
18.6652
18.1369
17.5004
150
100
50

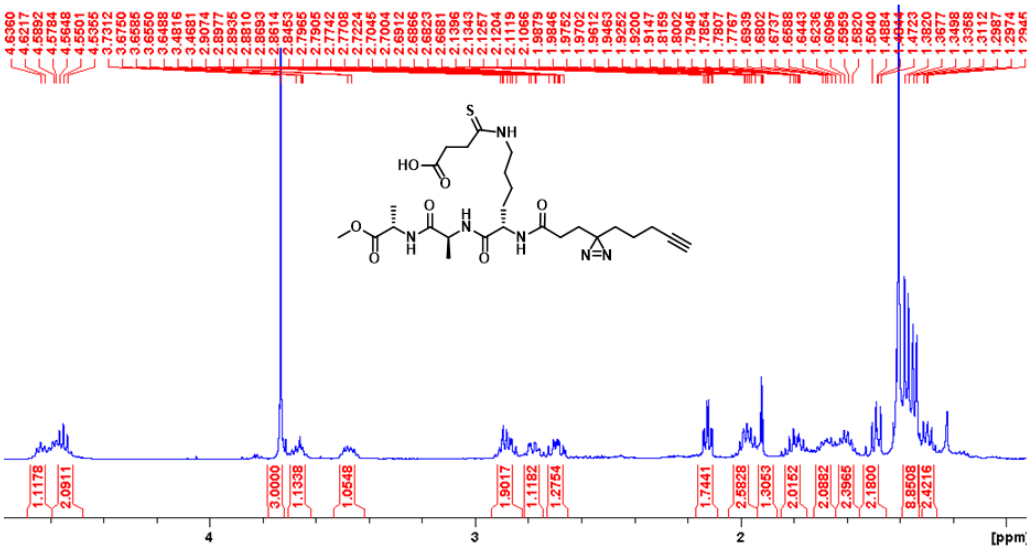
4.6360
4.6217
4.5892
4.5784
4.5648
4.5501
4.5355
3.7312
3.6750
3.6585
3.6550
3.6488
3.4816
3.4681
2.9074
2.8977
2.8935
2.8810
2.8693
2.8614
2.8453
2.7965
2.7905
2.7742
2.7708
2.7224
2.7045
2.7004
2.6912
2.6866
2.6823
2.6681
2.1396
2.1343
2.1257
2.1204
2.1119
2.1066
1.9879
1.9846
1.9752
1.9702
1.9612
1.9463
1.9252
1.9200
1.9147
1.8159
1.8002
1.7945
1.7854
1.7807
1.7767
1.6939
1.6802
1.6737
1.6588
1.6443
1.6236
1.6096
1.5959
1.5820
1.5040
1.4884
1.4723
1.3820
1.3677
1.3498
1.3358
1.3112
1.2987
1.2974
S
NH
HO
O
O
N
H
O
H
N
O
N
H
O
N=N
1.1178
2.0911
3.0000
1.1338
1.0548
1.9017
1.1182
1.2754
1.7441
2.5828
1.3053
2.0152
2.0882
2.3965
2.1800
8.8508
2.4216
4
3
2
[ppm]

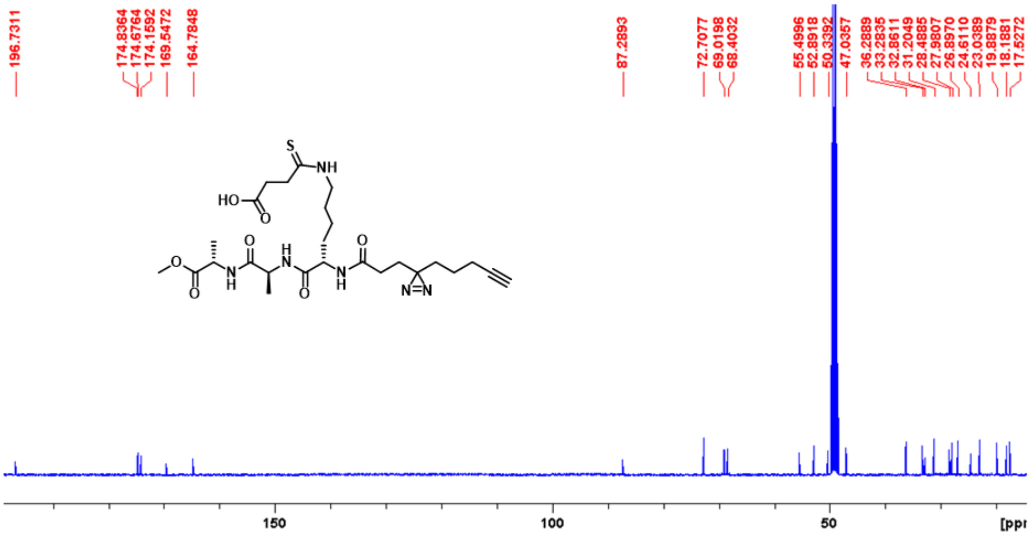
196.7311
174.8364
174.6764
174.1592
169.5472
164.7848
87.2893
72.7077
69.0198
68.4032
55.4996
52.8918
50.3392
47.0357
36.2889
33.2835
32.8611
31.2049
28.4885
27.9807
26.8970
24.6110
23.0389
19.8879
18.1881
17.5272
S
NH
HO
O
O
N
H
O
H
N
O
N
H
O
N=N
150
100
50

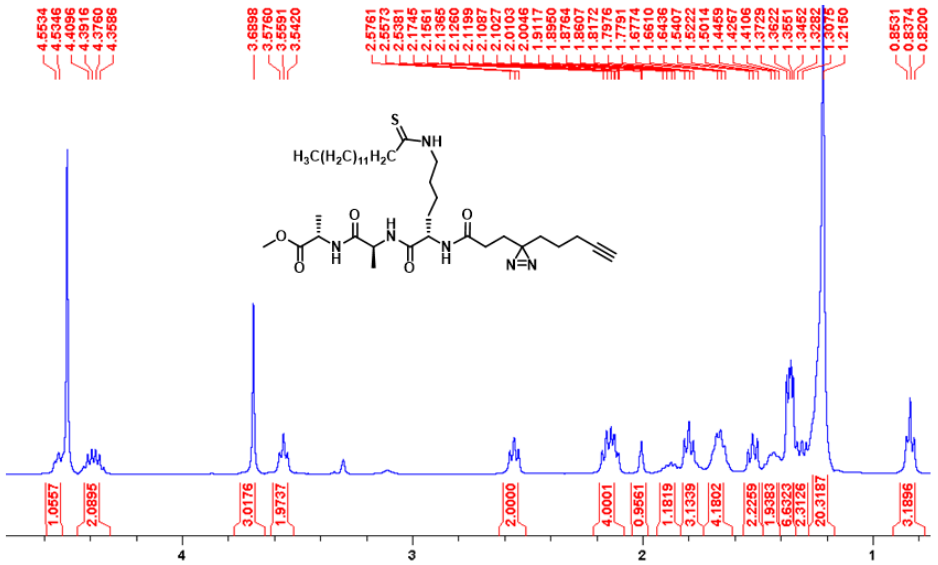

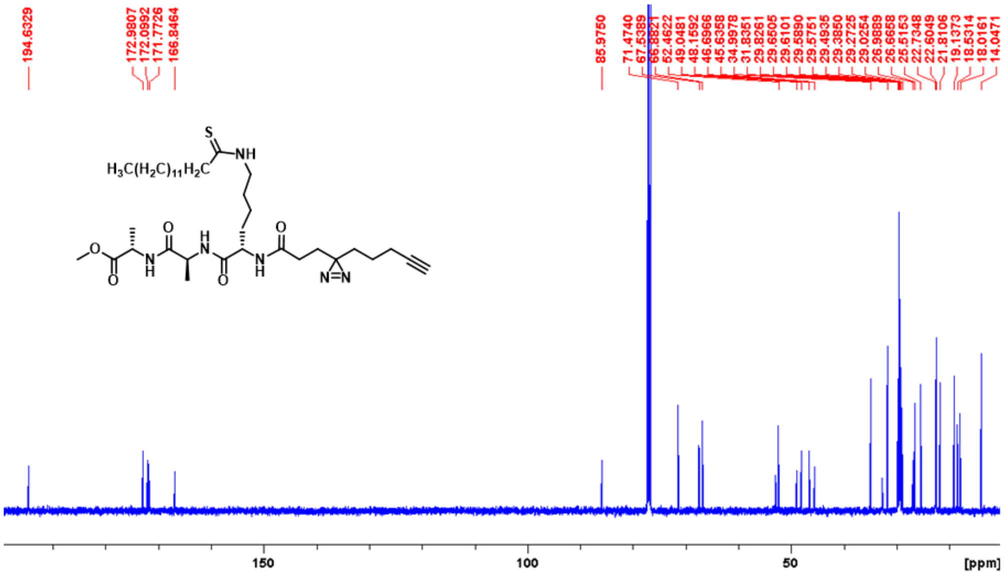

Supplement: Supplementary file 1 [file molecules-26-00011-s001.pdf]
